# Clinical evaluation of efficacy of intralesional platelet-rich plasma injection versus 1064 nm long-pulsed Neodymium:YAG laser in the treatment of inflammatory acne vulgaris in adolescent and post-adolescent patients: a prospective randomized split-face comparative study

**DOI:** 10.1007/s10103-022-03510-6

**Published:** 2022-01-27

**Authors:** Nayera Hassan Moftah, Aya Muhammad Mansour, Shady Mahmoud Attia Ibrahim

**Affiliations:** 1 Faculty of Medicine for Girls, Al-Azhar University, Cairo, Egypt; 2 Faculty of Medicine, Al-Azhar University, Cairo, Egypt

**Keywords:** Acne vulgaris, Platelet-rich plasma, Long-pulsed Nd:YAG laser, Inflammatory acne, Post-adolescent acne

## Abstract

Large numbers of local and systemic therapies are available for acne treatment. Common oral or topical retinoids, antibiotics, or keratolytics are used but sometimes are inconvenient, and side effects caused by these conventional therapies prompted a search for effective and safe treatments. This study aimed to evaluate the efficacy of intralesional platelet-rich plasma injection versus 1064 nm long-pulsed Nd:YAG laser in the treatment of moderate inflammatory acne vulgaris in both adolescents and post-adolescent patients. This split-face comparative study was carried out on thirty patients who suffered from moderate inflammatory and non-inflammatory acne vulgaris. The patients were classified into two groups: group I: adolescent (≤ 25 years) and group II: post-adolescent (< 25 years). Each group received four sessions of intralesional PRP injection on one side of the face and a long-pulsed Nd:YAG (1064 nm) laser on the other side with 2 weeks interval. Evaluation was done by blinded dermatologists using photographs and lesions counting and by patient satisfaction. Side effects were also noted. Both groups (adolescents and post-adolescent) showed a high statistically significant improvement of inflammatory as well as non-inflammatory lesions either in PRP or Nd:YAG laser–treated side with no significant difference between the two sides. The intralesional PRP injection and 1064 nm long-pulsed Nd:YAG laser are safe and effective methods for controlling inflammatory as well as non-inflammatory acne vulgaris in both adolescents and post-adolescent patients.

## Introduction

Acne vulgaris is a common disorder of the pilosebaceous unit, affecting about 85% of persons 12 to 25 years of age [1]. It often persists into adulthood, in 12–14% of cases with psychological, social, and emotional impairments [2].

Such a condition affecting wide range of population and leaving a permanent sequel needs effective management strategy that targets multiple pathogenic factors. Unfortunately despite that there are many modalities used for acne treatment, we are still suffering from low compliance of the patients, obvious adverse effects, and high rate of recurrence which limit their use. Therefore, increasing the armamentarium of non-pharmacologic treatment for acne vulgaris with safe and effective options is mandatory.

Recently, laser therapy has been widely used in the treatment of acne vulgaris due to its effectiveness and safety as it provides a more rapid response with less rate of recurrence, specifically 1064 nm Nd:YAG laser that has been documented in many studies [3, 4].

Regardless of the effect of PRP on tissue regeneration, it had been proved that the application of PRP showed a significant decrease in bacterial growth after 8 h for *S. aureus*, *S. epidermidis*, *methicillin-resistant S. aureus* (MRSA), and *Propionibacterium acnes* [5].

Many studies were conducted to evaluate PRP in the treatment of atrophic post-acne scars. An interesting finding was that all active acne lesions were cured after the PRP injection in acne scars concomitant with active acne [6]. This proves the anti-inflammatory and antimicrobial effect of PRP and suggests its possible role as an alternative modality in the treatment of inflammatory acne.

This study aimed to evaluate the efficacy of intralesional platelet-rich plasma injection versus 1064 nm long-pulsed Nd:YAG laser in the treatment of moderate inflammatory acne vulgaris in both adolescent and post-adolescent patients.

## Patients and methods

### Study design and subjects

A prospective randomized split-face comparative study was carried out on thirty patients collected randomly from the outpatient clinic of Dermatology and Venereology Department et al.-Zahraa University Hospital, Faculty of Medicine for Girls, Al-Azhar University and Al-Hussien University Hospital, Faculty of Medicine, Al-Azhar University, Cairo from September 2020 till May 2021. Approval from Research Ethics Committee of Faculty of Medicine for Girls, Al-Azhar University was obtained for the study protocol. Informed written consent has been obtained from the thirty patients before enrollment to participate and to use their photographs for scientific purposes.

All patients aged more than 18 years with active moderate inflammatory acne vulgaris including the inflammatory papules and nodules that count from 6 to 20 lesions in each half of the face according to Hayashi score [7].

Patients of both sexes and all skin phototypes were included. Exclusion criteria include the patients aged below 18 years old, pregnant or lactating women, patients with a history of coagulation disorders or receiving anticoagulant therapy, patients with hormonal disturbance or who receive oral contraceptive pills or hormonal therapy, patients on systemic or topical retinoids or prior intake in the previous 6 months, and patients of chronic diseases; patients with abnormal CBC findings (hemoglobin less than 10 g/dl or platelet less than 150 thousand/cmm) also were excluded. All patients with mild pure comedonal acne, pustular lesions, or severe nodulocystic acne, also patients with a skin infection or skin cancer in addition to patients who suffer from photosensitivity, had a history of keloidal scarring, and patients with unrealistic expectations had been excluded from the study.

### Treatment sessions

The patients were classified into two groups, group I: adolescent (age ≤ 25 years) and group II: post-adolescent (age < 25 years). Each group of the patients (groups I and II) received four sessions, 2 weeks apart of intralesional PRP injection on one side of the face (PRP-treated side) and long-pulsed Nd:YAG (1064 nm) laser on the other side (laser-treated side).

Each side was randomly selected by choosing a sealed opaque envelope containing a card labeled with either laser or PRP injection represented the treatments for right and left split face side. Topical anesthetic cream was applied for 30 min before the session (Pridocaine®; a mixture of Lidocaine 25% and Prilocaine 25%, made in Egypt).

- Long-pulsed Nd:YAG (1064 nm) laser (DEKA, Motus AY, Italy) performed on one side (laser side); the lesional area had been treated with three passes and single pass over the perilesional area with the following settings: fluence 35 J/cm^2^, pulse duration 30 ms, spot size 10 mm. Eye protection was provided by using stainless steel scleral shields. The patients were advised to apply topical sunscreen daily.
- PRP preparation and intralesional injection: 10 mL of venous blood had been collected from the antecubital vein under complete aseptic conditions in tubes containing sodium citrate 3.2% as an anticoagulant (sodium citrate 9NC, VACO MED) then subjected to the double spin method. The first one was slow at 3000 rpm for 7 min then the second centrifugation was faster at 4000 rpm for 5 min [8]. The resultant plasma was subsequently aspirated and activated by calcium chloride (CaCl_2_) in the proportion of 0.1 mL of CaCl_2_ per 0.9 mL of PRP, thus obtaining a concentration of activated PRP.

The injection was done by a 30 gauge needle, with a maximum of 1 mL/session.

### Assessment of the efficacy of the treatment

The evaluation was done before the treatment at baseline and before each treatment session and 1 month after the last session by photographs using the digital camera (Nikon Coolpix L340, 20.2megapixels, made in China), lesion counting and grading according to Hayashi score by blinded dermatologist to document improvement and to detect any possible side effects.

The expected side effects were as follows:

- Persistent erythema (> 48 h); graded using a 3-point scale (the 0 level is defined as “No erythema,” while a level of 3 is defined as “severe erythema”).
- Post-laser or PRP injection hyperpigmentation; graded as 0 = no hyperpigmentation and 1 = presence of hyperpigmentation.
- Post-laser or PRP injection hypopigmentation; graded as 0 = no hypopigmentation and 1 = presence of hypopigmentation.
- Bruises; graded as 0 = no bruises and 1 = presence of bruises.

The patients were reevaluated 2 months after the treatment to detect recurrence (reappearance of the same lesions after complete cure or appearance of new lesions) and to record patient satisfaction by a 10-point visual analog scale (VAS, 0–10; the 0 level is defined as “not satisfied,” while a level of 10 is defined as “completely satisfied”).

### Statistical analysis

The sample size was calculated using the following formula [9]:

$n=\frac{2{\mathrm{SD}}^{2}\left(Z\alpha +Z\beta \right)}{{d}^{2}}$ (*n* = sample size, SD = standard deviation, *Zα* = 1.96, *Zβ* = 0.84, *d* = μ_2_-μ_1_

According to a previous study by Mohamed et al. [10], who stated that, the Nd:YAG–treated side showed a reduction in the count of inflammatory and non-inflammatory lesions counts by 70.2% and 17.9%, respectively.

So, the sample size = $\left(\frac{{\left(2\times 12\right)}^{2}{\left(1.96+0.84\right)}^{2}}{{\left(19.1-4.2\right)}^{2}}\right)=20.3$

The minimum sample size needed for this study was found to be 20 patients.

Thirty patients had been included with a 30% increment due to the possibility of lost patients during follow-up.

Data were fed to the computer and analyzed using the SPSS program version 23 (SPSS Inc., Chicago, IL, USA). The comparison between two paired groups with quantitative data and parametric distribution was done by using ***Paired t-test.*** Also, the comparison between two independent groups with quantitative data and parametric distribution was done by using an ***Independent t-test.*** P < 0.05 value was set as statistically significant.

## Results

Thirty patients participated in the study, and all the patients suffered from moderate inflammatory acne vulgaris with erythematous papules and nodules with ages ranged from 18 to 40 years (mean age ± SD = 23.73 years ± 5.77); they were 26 females (86.7%) and 4 males (13.3%).

They classified according to their age into group I: adolescent (age ≤ 25 years), 17 patients (56.7%) and group II: post-adolescent (age < 25 years), 13 patients (43.3%).

Only 8 patients (26.7%) reported a negative family history of acne vulgaris; while 22 patients (73.3%) reported a positive family history.

According to skin type, 9 patients (30%) were skin type III, 16 patients (53.3%) were skin type IV, and 5 patients (16.7%) were skin type V according to Fitzpatrick’s skin type classification.

**Fig. 1:**
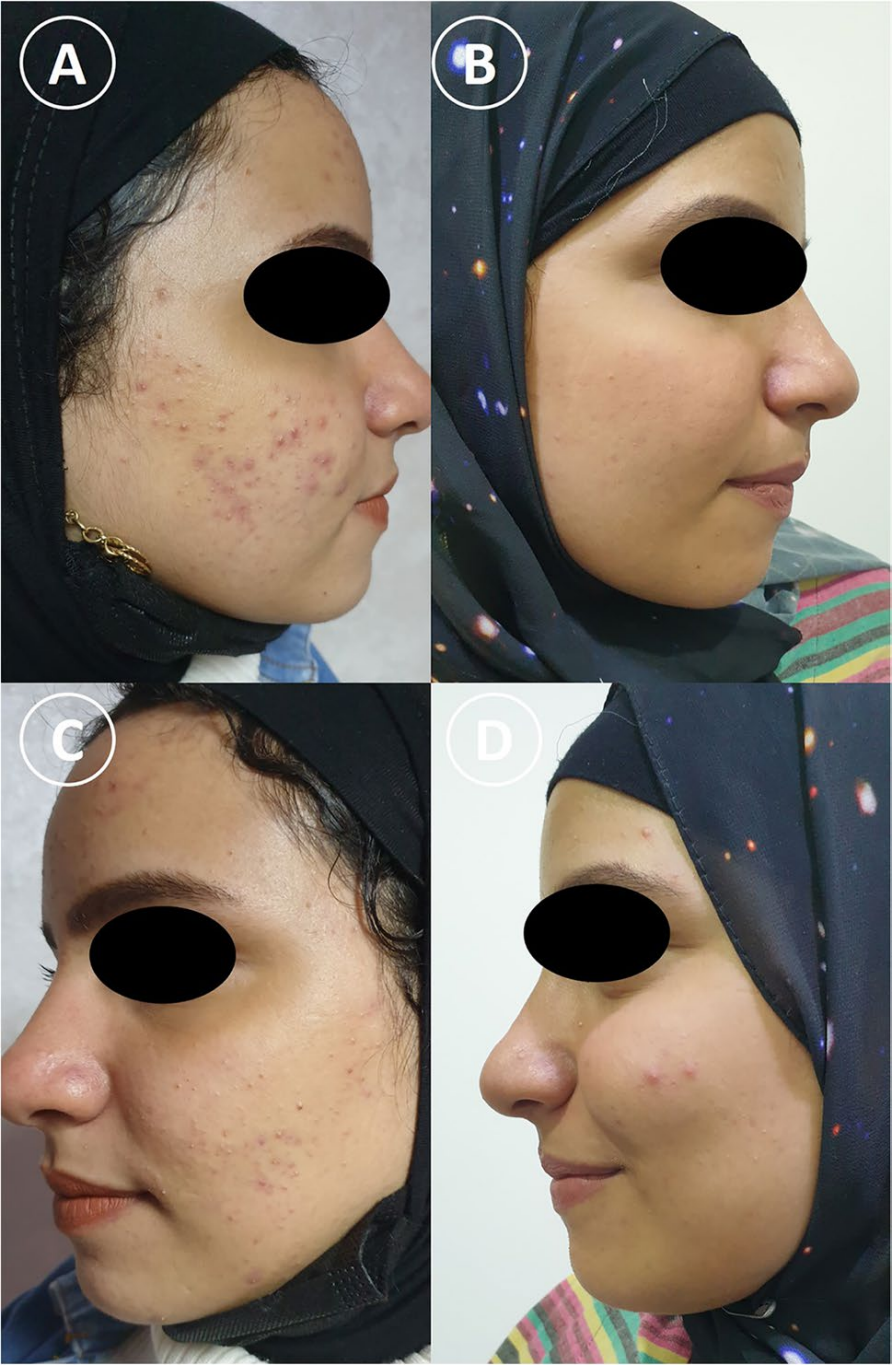
A 22-year-old female skin type IV: **a** and **b** PRP-treated side of the face **a** before treatment, **b** after treatment. **c** and **d** Laser-treated side by 4 Nd:YAG treatment sessions, **c** before treatment, **d** after treatment. Clinical improvements were observed on both sides

**Fig. 2:**
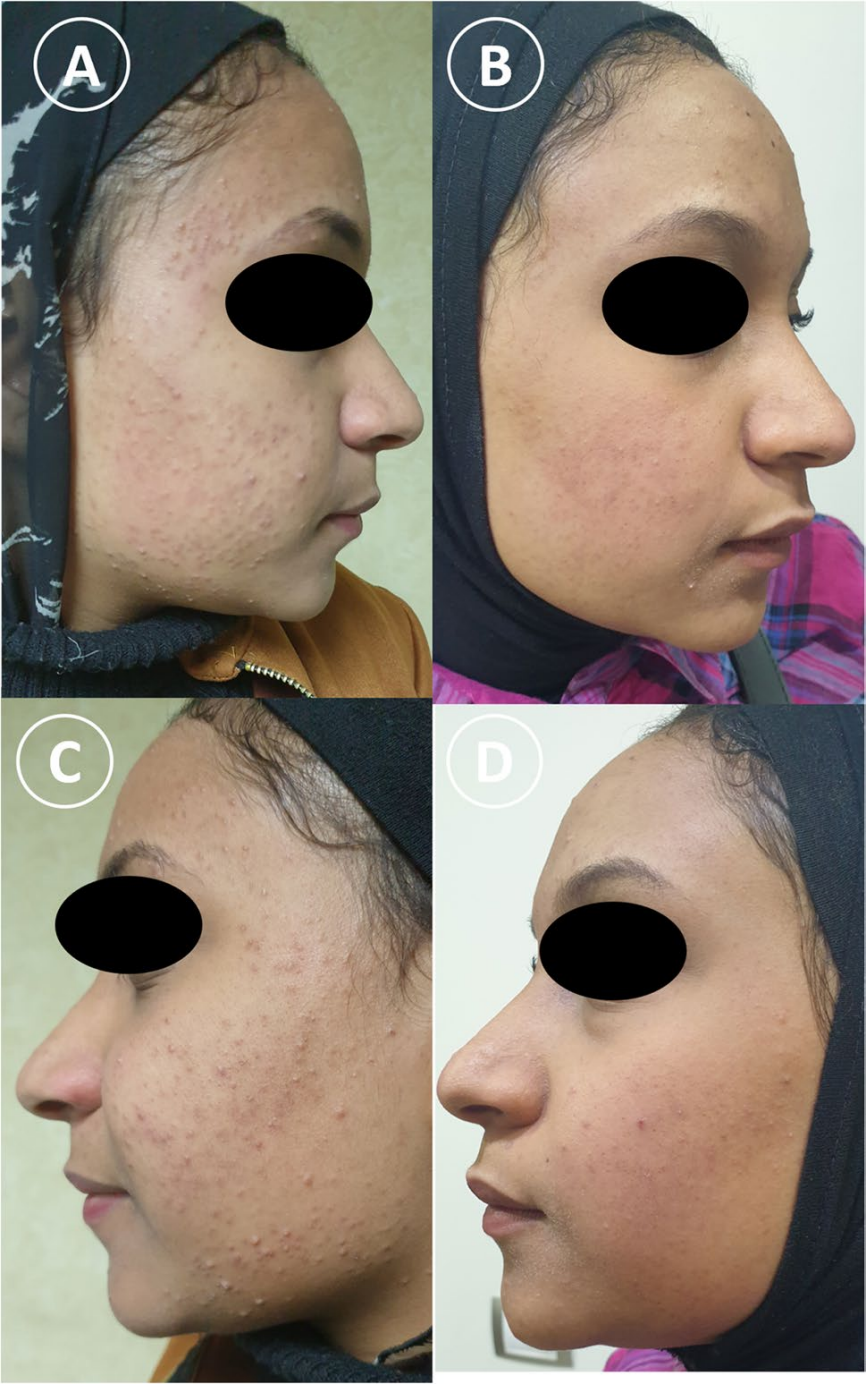
A 19-year-old female skin type V: **a** and **b** PRP-treated side of the face **a** before treatment, **b** after treatment. **c** and **d** Laser-treated side by 4 Nd:YAG treatment sessions, **c** before treatment, **d** after treatment. Clinical improvements were observed on both sides

There was a statistically significant decrease in the number of inflammatory lesions in the PRP-treated side after the 4th session by a percentage of 58.77% ± 14.98 compared with that at baseline with a *P* value < 0.001. Also, there was a statistically significant decrease in the number of lesions in Nd:YAG laser–treated side after the 4th session by a percentage of 55.47% ± 17.53 compared with that at baseline with a *P* value < 0.001 (Figs. 1 and 2). Regarding the non-inflammatory lesions, there was a statistically significant improvement in PRP-treated side after treatment compared with that at baseline by a percentage of 47.86% ± 18.91 and with a *p*-value < 0.001. Also, there was a statistically significant decrease in the non-inflammatory lesions in the laser-treated side after treatment compared with that at baseline by a percentage of 47.48% ± 16.08 and with a *p*-value < 0.001 (Table 1).

**Table 1:** Comparison of dermatologist evaluation of mean number and percentage of improvement of inflammatory and non-inflammatory lesions of PRP-treated side and Nd:YAG laser–treated side

		Before treatment	After treatment	t*	*P*-value	Percentage of improvement after treatment
		No. = 30	No. = 30			
Inflammatory lesions						
PRP-treated side	Mean ± SD Range	12.47 ± 3.28 8–20	5.11 ± 2.04 2–10	− 12.926	< 0.001*	58.77 ± 14.98 27.27–84.21
Nd:YAG laser–treated side	Mean ± SD Range	11.27 ± 3.52 5–19	5 ± 2.42 2–10	− 11.058	< 0.001*	55.47 ± 17.53 20–81.82
*t*1		**1.366**	**0.179**			**0.756**
*P*1 value		**0.177**	**0.859**			**0.453**
Non-inflammatory lesions						
PRP-treated side	Mean ± SD Range	12.13 ± 6.32 4–25	6.3 ± 4.13 2–16	− 7.740	< 0.001*	47.86% ± 18.91 12.5–90.48
Nd:YAG laser–treated side	Mean ± SD Range	11.6 ± 5.61 3–24	6.07 ± 3.47 1–13	− 8.584	< 0.001*	47.48% ± 16.08 15.38–83.33
*t*1		**0.346**	**0.237**			**0.082**
*P*1 value		**0.731**	**0.814**			**0.935**

*P* comparison between before and after treatment; *P*1 comparison between PRP-treated side and Nd:YAG laser–treated side

^*^P-value < 0.05 is considered statistically significant; *P* value > 0.05 is considered statistically non-significant

t*: Paired *t*- test

*t*1: Independent *t*-test

There was no statistically significant difference between PRP-treated side and Nd:YAG laser–treated side in the lesion count before treatment and after treatment and percentage of improvement either for inflammatory or non-inflammatory lesions (Table 1).

There was a statistically significant difference found between the percentage of improvement of the inflammatory and non-inflammatory lesions in the PRP-treated side; the inflammatory lesions have a higher improvement with a *p*-value = 0.026 while there was no statistically significant difference between the two types of lesions at the laser-treated side (Table 2).

**Table 2:** Comparison between PRP-treated side and Nd:YAG laser–treated side regarding the percentage of improvement of inflammatory lesions and non-inflammatory lesions after the treatment

Percentage of improvement after treatment		PRP-treated side	Laser-treated side
		No. = 30	No. = 30
Inflammatory lesions	Mean ± SD	58.77% ± 14.98	55.47% ± 17.53
	Range	27.27–84.21	20–81.82
Non-inflammatory lesions	Mean ± SD	47.86% ± 18.91	47.48% ± 16.08
	Range	12.5–90.48	15.38–83.33
t*		**2.355**	**1.970**
*P*-value		0.026*	**0.059**

^*^P-value < 0.05 is considered statistically significant; *P*-value > 0.05 is considered non-significant (NS)

^*^Paired *t*-test

In group I (adolescents), the percentage of improvement of the inflammatory lesions was higher than the non-inflammatory lesions, either in PRP or laser-treated side with a statistically significant difference (*p*-value = 0.007 and 0.002 for PRP- and laser-treated sides, respectively) but there was no statistically significant difference found between the percentage of improvement of both types of lesions in group II (post-adolescent) (Table 3).

**Table 3:** Comparison between group I (adolescent) and group II (post-adolescent) regarding the percentage of improvement of inflammatory lesions and non-inflammatory lesions in PRP- and Nd:YAG laser–treated side

% of improvement after treatment in PRP-treated side		Group I: adolescent (age < 25 years)	Group II: post-adolescent (age ≥ 25 years)	*t*•		*P*-value
		No. = 17	No. = 13			
Inflammatory lesions	Mean ± SD	64.23% ± 12.78	52.47% ± 15.29	**2.217**		0.036*
	Range	33.33–84.21	27.27–80			
Non-inflammatory lesions	Mean ± SD	46.33% ± 22.20	49.85% ± 14.13	** − 0.500**		**0.621**
	Range	12.5–90.48	20–71.43			
t*		**3.133**	**0.418**			
*P*1-value		0.007*	**0.684**			
% of improvement after treatment in Nd:YAG laser–treated side		Group I: adolescent (age < 25 years)	Group II: post-adolescent (age ≥ 25 years)	*t*•	*P*-value	
		No. = 17	No. = 13			
Inflammatory lesions	Mean ± SD	61.25% ± 16.45	48.81% ± 16.92	**1.970**	**0.060**	
	Range	23.08–81.82	20–73.33			
Non-inflammatory lesions	Mean ± SD	44.04% ± 17.53	51.99% ± 13.27	** − 1.362**	**0.184**	
	Range	15.38–83.33	30–75			
t*		**3.913**	** − 0.788**			
*P*1-value		0.002*	**0.446**			

*P* = comparison between adolescent and post-adolescent; *P*1 = comparison between PRP-treated side and Nd:YAG laser–treated side

^*^P-value < 0.05 is considered statistically significant; *P*-value > 0.05 is considered non-significant (NS)

^*^Paired *t*-test

•: Independent *t*-test

In the PRP-treated side, there was statistically significant difference between group I (adolescents) and group II (post-adolescent) in the percentage of improvement of inflammatory lesions after the 4th session; it was higher in group I (adolescents) with *P*-value = 0.036 but there was no statistically significant difference between two groups regarding the percentage of improvement in non-inflammatory lesions. Also, in the laser-treated side, there was no statistically significant difference between group I (adolescents) and group II (post-adolescent) regarding percentage of improvement of either inflammatory or non-inflammatory lesions (Table 3).

The patients were asked about their satisfaction towards each treatment modality using a 10-point visual analog scale and their satisfaction was higher for PRP than Nd:YAG laser (8.36 ± 1.87 and 7.89 ± 2.02, respectively) but with no statistically significant difference between modalities (Table 4).

**Table 4:** Comparison between PRP-treated side and Nd:YAG laser–treated side regarding patients’ satisfaction using a 10-point visual analog scale

Patients’ satisfaction	PRP-treated side	Laser-treated side	Test value•	*P*-value
	No. = 30	No. = 30		
Mean ± SD	8.36 ± 1.87	7.89 ± 2.02	0.891	0.377
Range	4 – 10	4 – 10		

*P*-value > 0.05 is considered non-significant (NS); *P*-value < 0.05 is considered significant (S)

•: Paired *t*- test

At 8 weeks after the 4th session, during the recording of the secondary efficacy outcome, both methods showed high statistical improvement by a percentage of 63.70% (± 21.12) and 61.53% (± 20.10) for PRP and Nd:YAG laser respectively with no statistically significant difference between the two lines of treatment.

Recurrence occurred in a mild degree only in 6 patients (20%), 4 patients from group I (adolescents) and 2 patients from group II (post-adolescent).

Regarding the side effects, no side effects were recorded in this study or reported by the patients except for only one patient who developed vasovagal syncope during venipuncture of the blood sample for PRP preparation. The pain was extremely tolerable especially with the usage of the topical anesthetic cream. Also, erythema and edema after injection were mild and resolved within few hours after the session, and careful injection prevents the occurrence of bruises.

No inflammation or allergic reactions were noticed after PRP injection except for only one patient who developed contact dermatitis from the anesthetic cream.

Regarding the Nd:YAG laser treatment, no side effects were reported but only one male patient was annoyed about the hair reduction occurred in the beard area. Otherwise, no post-laser hyperpigmentation or hypopigmentation had been documented.

## Discussion

Due to sometimes failures of conventional treatments of acne vulgaris, antibiotic resistance, or unsuitability of pharmacotherapy for some patients, there was a great need to develop new therapeutic options especially laser therapy which gained wide popularity due to its effectiveness, convenience, and safety [11].

The efficacy of 1064 nm long-pulsed Nd:YAG laser for acne vulgaris and acne scars was proved [4, 12]. Platelet-rich plasma (PRP) is effective in treatment of acne scars. However, its effect on active acne was reported with fractional erbium laser [13]. No study compared its effect with Nd:YAG laser in treatment of acne vulgaris.

In the present study, there was a statistically significant difference between both groups regarding non-inflammatory lesions before treatment as it was higher in group I (adolescents).

This is in agreement with a study by Goulden et al. [14] who studied the clinical features of post-adolescent acne and reported that post-adolescent acne is consisting predominantly of inflammatory lesions.

In this study, the laser-treated side showed a high statistically significant decrease in the number of inflammatory lesions after the 4th session by a percentage of 55.47% ± 17.53 and in the non-inflammatory lesions by a percentage of 47.48% ± 16.08.

These results were in keeping with a study by Mohamed et al. [9] a split-face study with 74 patients suffered from facial acne, and the Nd:YAG–treated side showed acne lesion counts improvement by 70.2% for inflammatory and 17.9% for non-inflammatory lesions after three treatment sessions, 4-week interval with spot size 15 mm, pulse duration 20 ms, and fluence 30–35 J. Also, Monib et al. [15] which compared 1064 nm long-pulsed Nd:YAG laser versus IPL in inflammatory and non-inflammatory acne. The Nd:YAG group improved by a percentage of 65.7% for inflammatory and 44% for non-inflammatory lesions after three treatment sessions, 2-week interval with spot size 7 mm, pulse duration 40 ms, and fluence 40–50. Furthermore, Chalermsuwiwattanakan et al. [3] compared 1064 nm long-pulsed Nd:YAG laser and 595-nm pulsed dye laser for the treatment of acne vulgaris; 34 patients involved in the study and there was a significant improvement of inflammatory and non-inflammatory lesions by 50.06% and 15.95%, respectively, after three treatment sessions by the long-pulsed Nd:YAG laser of 2-weeks with a spot size of 7 mm, pulse duration of 5 ms, and fluence of 30 J/cm^2^ [3].

The different responses between these study results and the current study may be due to the difference in the patient characteristics, Nd:YAG parameters, manufacture of laser device, number of treatment sessions, and the duration between sessions.

The therapeutic effects of 1064 nm Nd:YAG laser depends on its effect on the vascular component of inflammatory acne in addition to the alteration of cytokine release, including the upregulation of TGF-β and the downregulation of IL-8 and TLR-2. The improvement of non-inflammatory acne might be due to the thermal damage to sebaceous glands causing a reduction in sebum production [3].

In the current study, in the PRP-treated side, there was a highly statistically significant decrease in the number of inflammatory lesions after the 4th session by a percentage of 58.77% ± 14.98 and the non-inflammatory lesion by a percentage of 47.86% ± 18.91.

There is only one study by Ibrahim et al. [5] which investigated the intralesional PRP injection in facial acne in comparison with topical erythromycin and showed that there was a statistically significant difference in the PRP group before and after treatment in the number of papulopustular and nodulocystic lesions while no significant difference in the number of comedonal lesions.

This is similar to the present study regarding the significant improvement of the inflammatory lesions after the PRP injection, the high patient satisfaction, and low rate of recurrence. However, in the present study, there was a significant improvement in the non-inflammatory lesions by a percentage of 47.86% that may be due to different characteristics of the patients, method, and equipments of PRP preparation. The current results support the hypothesis that the inflammatory changes resulting from toll-like receptor activation and secretion of IL-1α from keratinocytes occurred early in the development of acne lesions and could be the initiating steps in comedogenesis [16] preceding the hyperproliferative changes so the PRP targeted the first step in acne due to its anti-inflammatory and antibacterial properties.

The anti-inflammatory effect of PRP injection was confirmed in a study by Ghoz et al. [17], through the immunohistochemical examination to detect NF-κB (nuclear factor kappa-light-chain enhancer of activated B cells), which markedly decreased after PRP injection when compared with its level before treatment; NF-κB is a transcription factor that upregulates many pro-inflammatory cytokines involved in acne pathogenesis. [18]

The antibacterial mechanisms of PRP may be associated with the antimicrobial peptides and other active substances that are released after activation and platelet degranulation as the human beta-defensin 2 (hBD-2). [16] It is present in healthy pilosebaceous unit to prevent microbial invasion and showed a marked upregulation by immunoreactivity in the biopsy of acne lesions and perilesional areas. [19] It also proved to be present in the PRP by immunohistochemistry and Western Blot in a concentration of 1786 pg/mL. [20]

The main aim of the study was to compare the efficacy of intralesional PRP injection versus long-pulsed 1064 nm Nd:YAG laser in moderate acne in the adolescent and post-adolescent patients and both methods showed high significant improvement as mentioned before with no statistically significant difference found between PRP-treated side and Nd:YAG laser–treated side and up to our knowledge, it could be the first study to compare any therapeutic procedure between adolescents and post-adolescent patients considering both types of acne lesions as follows:

In group I (adolescents), there was a statistically significant difference found between the percentages of improvement of both types of lesions; it was greater in inflammatory lesions that respond better than non-inflammatory lesions either for PRP- or Nd:YAG laser–treated sides, while in group II (post-adolescent), there was no statistically significant difference found between the percentage of improvement of both types of lesions either in PRP- or Nd:YAG laser–treated sides.

Regarding the percentage of improvement of inflammatory lesions after PRP injection, the percentage of improvement was greater in group I (adolescent) than group II (post-adolescent), indicating that both modalities are effective in the treatment of acne vulgaris with the maximum efficacy for PRP in treating young patients (adolescents) with prominent inflammatory lesions.

Eight weeks after the last sessions and despite the slight recurrence in small number of patients, only 2 patients in group II (post-adolescent) and 4 patients in group I (adolescents), the overall percentage of improvement increased than at 4 weeks denoting the prolonged effect of both PRP and Nd:YAG laser.

The high patient satisfaction also confirmed the efficacy of PRP and Nd:YAG laser as both methods provide a noticeable rejuvenation effect by improving skin texture and minimize post-acne complications as scarring, erythema, and hyperpigmentation.

The PRP injection is considered a promising and attractive strategy in acne treatment as it is minimally invasive, simple procedure and cost-effective that did not require expensive equipment, without any side effects on prolonged use. Also, the autologous property eliminates concerns about the risk for disease transmission or immunological reactions in addition to the proven efficacy in treating resistant tissue infections, modulating inflammation, and enhancing collagen remodeling and tissue regeneration [21]

The same for Nd:YAG laser as it was effective, relatively safe, tolerable, not time-consuming maneuver with limited downtime and minimal side effects [22]

## Conclusion

According to the present study results, both the intralesional PRP injection and 1064 nm long-pulsed Nd:YAG laser are safe and effective methods for controlling inflammatory as well as non-inflammatory acne lesions either in adolescent or post-adolescent patients.
